# Supplementary material for: A Green and Effective Polyethylene Glycols-Based Microwave-Assisted Extraction of Carnosic and Rosmarinic Acids from Rosmarinus officinalis Leaves
Source: Foods. 2023 Apr 24;12(9):1761. doi: 10.3390/foods12091761 (PMC10177924; doi:10.3390/foods12091761)
Supplement: Supplementary file 1 [file foods-12-01761-s001.zip › foods-2300410-supplementary.pdf]

A Green and Effective Polyethylene Glycols-Based Microwave-Assisted Extraction of Carnosic and Rosmarinic Acids from *Rosmarinus Officinalis* Leaves

Chunyan Zhu[1], Yunchang Fan[1,*] and Xiujun Bai[2]

[1] College of Chemistry and Chemical Engineering, Henan Polytechnic University, Jiaozuo 454003, China

[2] Shijiazhuang ENN gas Co., Ltd., Shijiazhuang, 050081, China

* Corresponding author. E-mail address: fanyunchang@hpu.edu.cn

Table S1. Results of regression analysis of the second-order polynomial models for the optimization of the extraction of CA and RA.

Term	Coefficients Estimated	*p*-Value	*t*-Value
		**RA**	
Intercept	1.34	< 0.0001*	25.56
A	−0.014	0.378	−0.92
B	−0.016	0.288	−1.12
C	0.016	0.300	1.09
D	0.046	0.008*	3.29
AB	0.0025	0.910	0.12
AC	0.018	0.439	0.81
AD	0.0058	0.796	0.27
BC	−0.038	0.111	−1.75
BD	−0.036	0.118	−1.71
CD	0.016	0.484	0.73
A^2	−0.026	0.366	−0.95
B^2	0.0052	0.872	0.17
C^2	−0.038	0.198	−1.38
D^2	−0.17	< 0.0001*	−5.56
		**CA**	
Intercept	2.64	< 0.0001*	17.14
A	0.22	0.001*	4.91
B	0.064	0.155	1.54
C	0.26	< 0.0001*	5.89
D	−0.077	0.092	−1.86
AB	−0.0078	0.905	−0.12
AC	−0.030	0.660	−0.45
AD	−0.16	0.031*	−2.52
BC	−0.12	0.086	−1.91
BD	−0.034	0.598	−0.55
CD	−0.028	0.666	−0.44
A^2	−0.42	< 0.0001*	−5.30
B^2	−0.17	0.095	−1.85
C^2	−0.45	< 0.0001*	−5.56
D^2	−0.21	0.045*	−2.29

*Significant ($p < 0.05$).

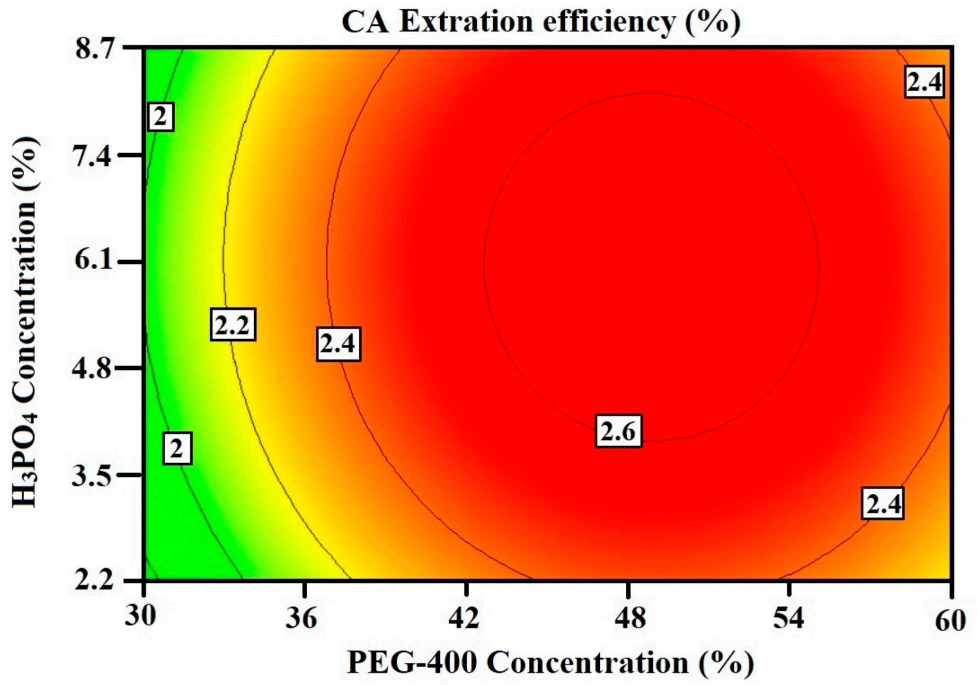

Figure S1. Contour plot of the interactive effects of PEG-400 concentration and H3PO4 concentration on the extraction of CA.

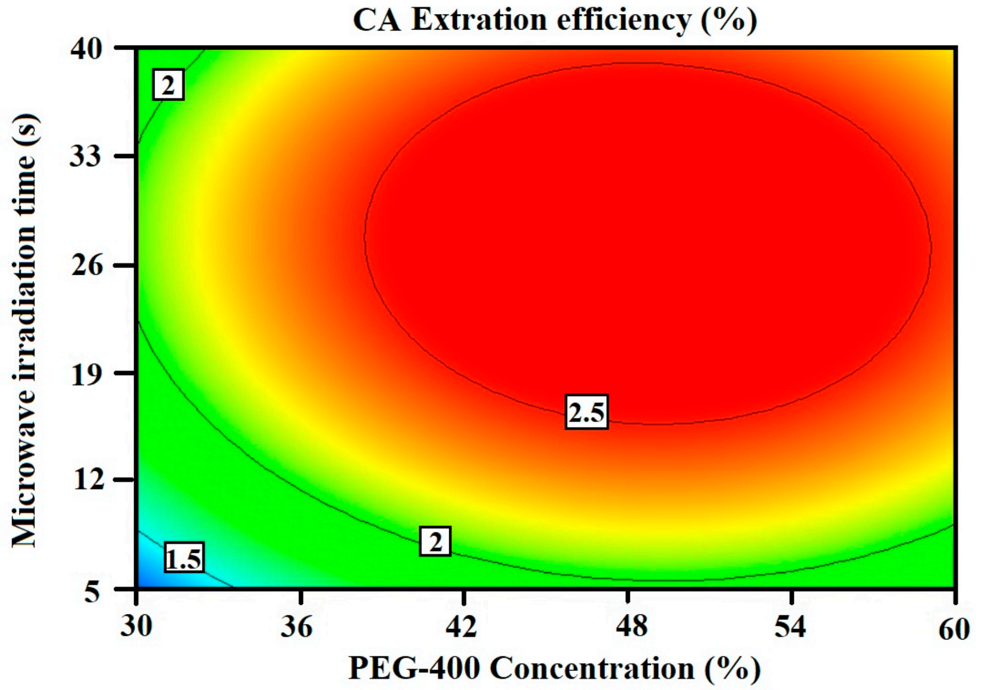

Figure S2. Contour plot of the interactive effects of PEG-400 concentration and microwave irradiation time on the extraction of CA.

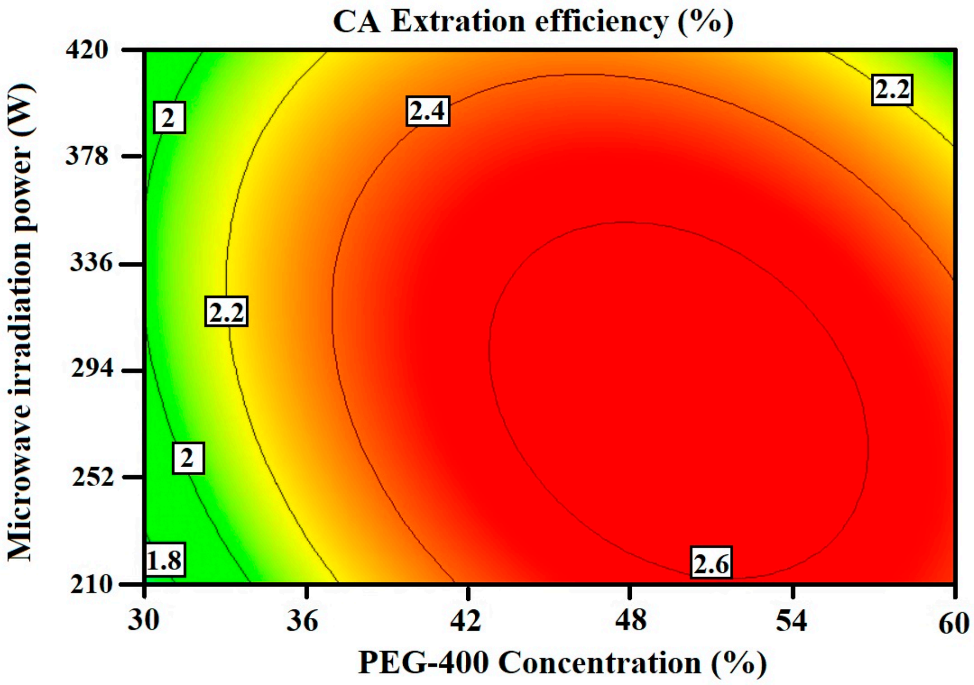

Figure S3. Contour plot of the interactive effects of PEG-400 concentration and microwave irradiation power on the extraction of CA.

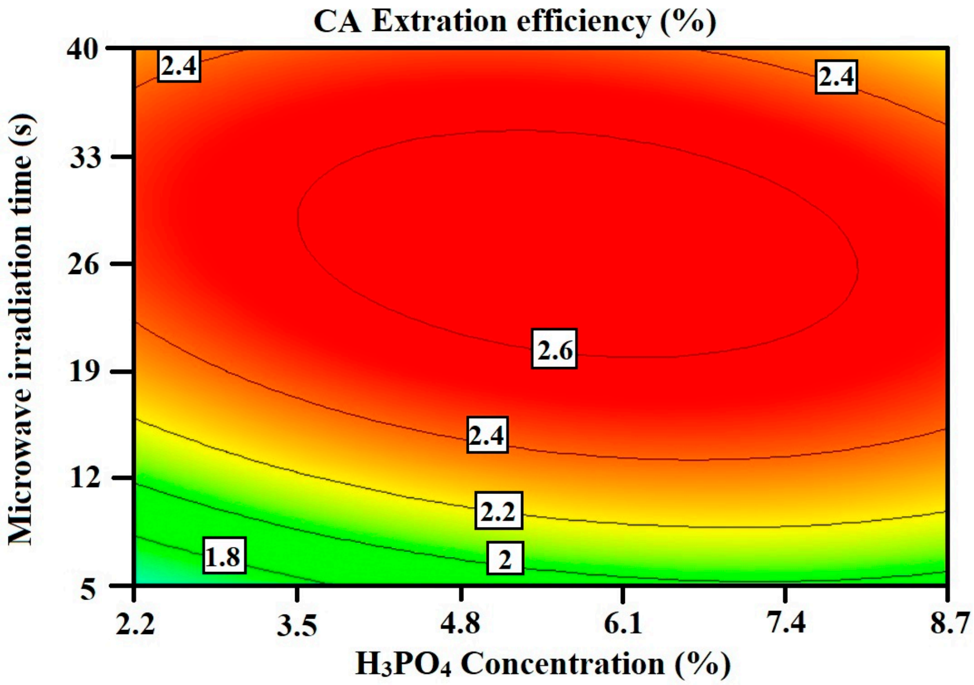

Figure S4. Contour plot of the interactive effects of H3PO4 concentration and microwave irradiation time on the extraction of CA.

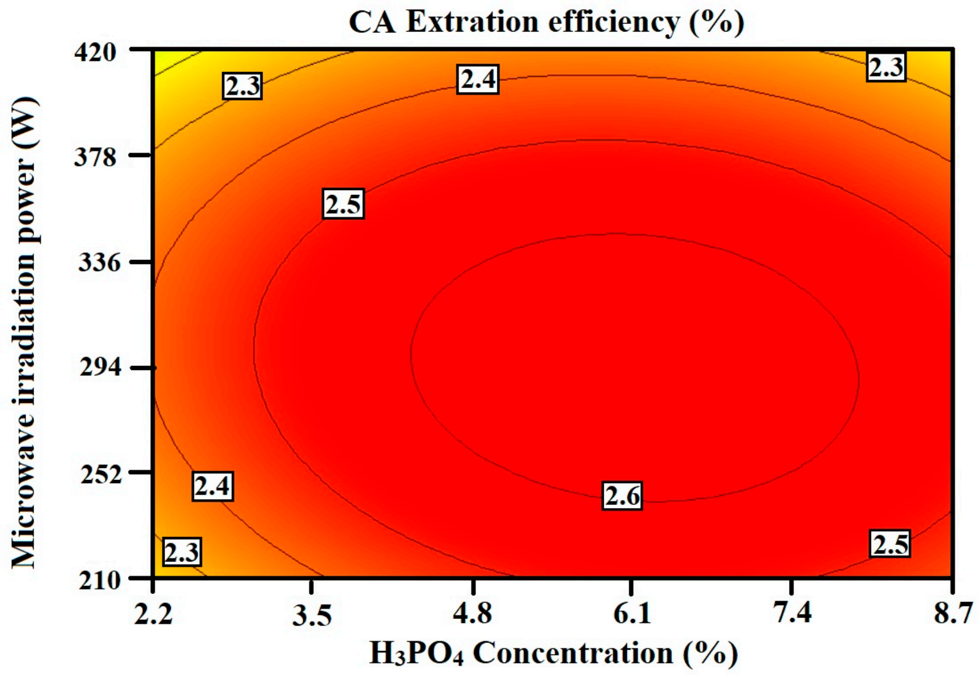

Figure S5. Contour plot of the interactive effects of H3PO4 concentration and microwave irradiation power on the extraction of CA.

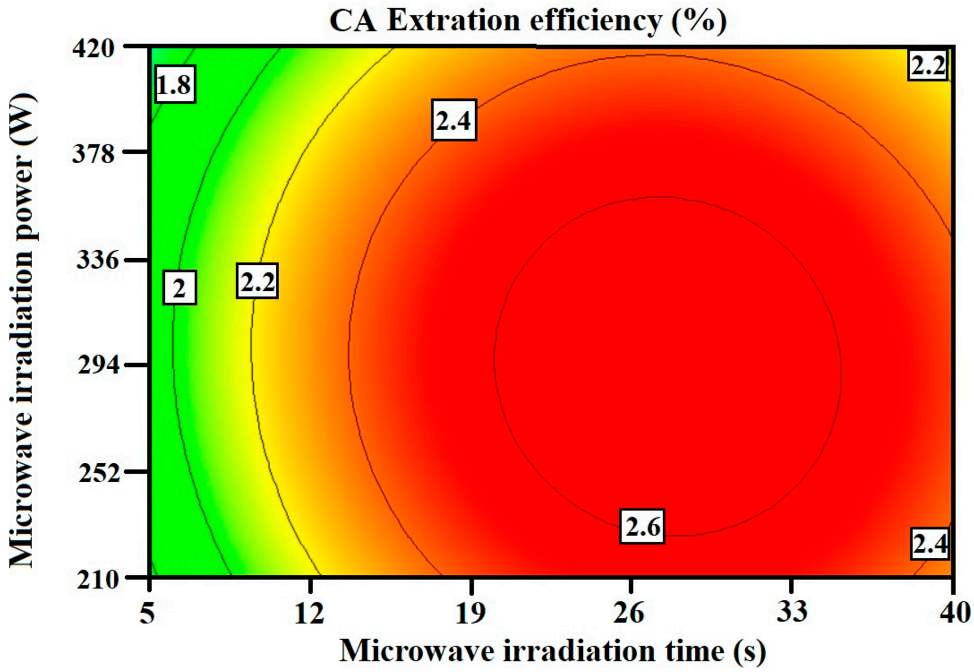

Figure S6. Contour plot of the interactive effects of microwave irradiation time and microwave irradiation power on the extraction of CA.

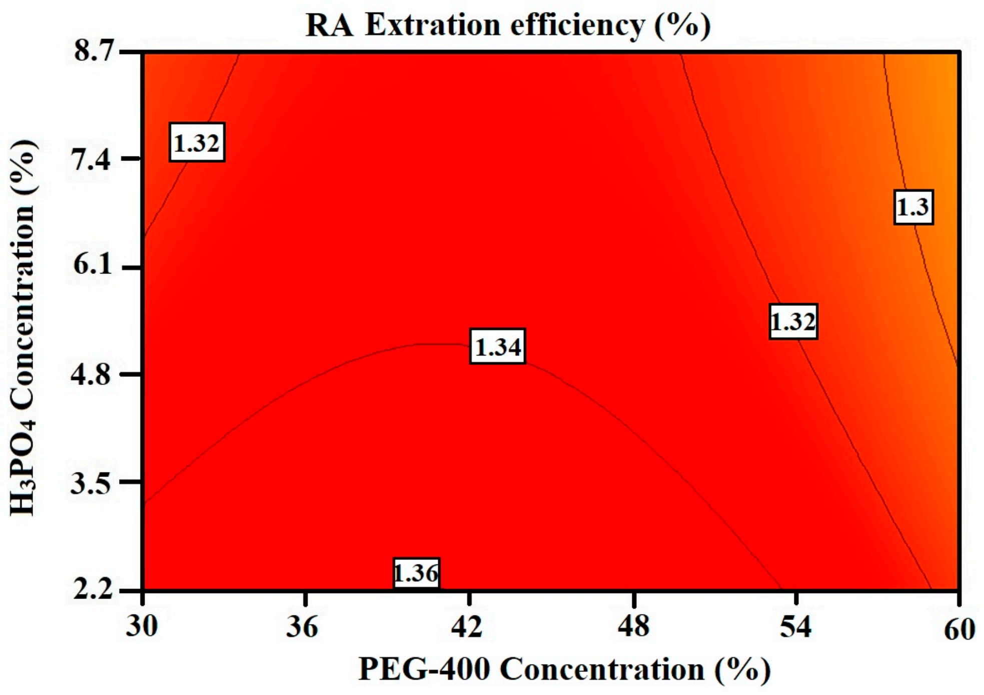

Figure S7. Contour plot of the interactive effects of PEG-400 concentration and H3PO4 concentration on the extraction of RA.

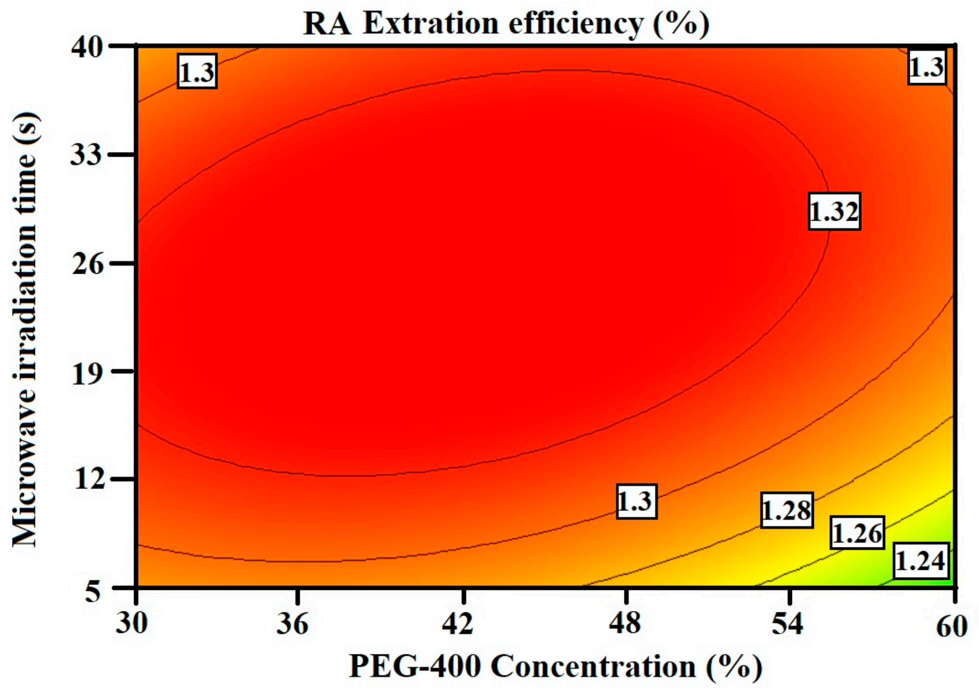

Figure S8. Contour plot of the interactive effects of PEG-400 concentration and microwave irradiation time on the extraction of RA.

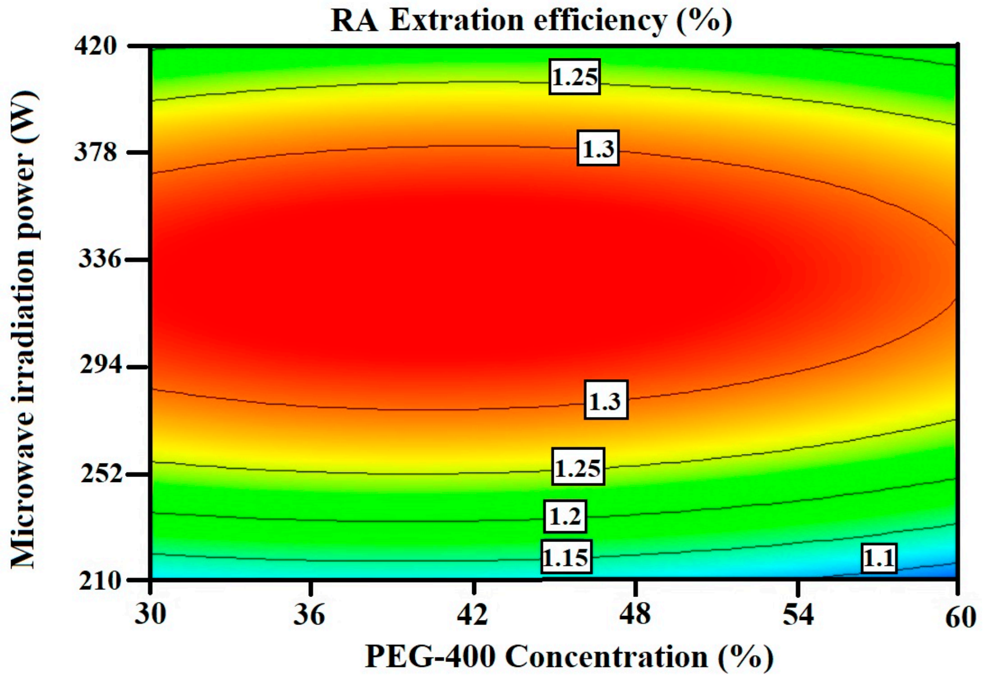

Figure S9. Contour plot of the interactive effects of PEG-400 concentration and microwave irradiation power on the extraction of RA.

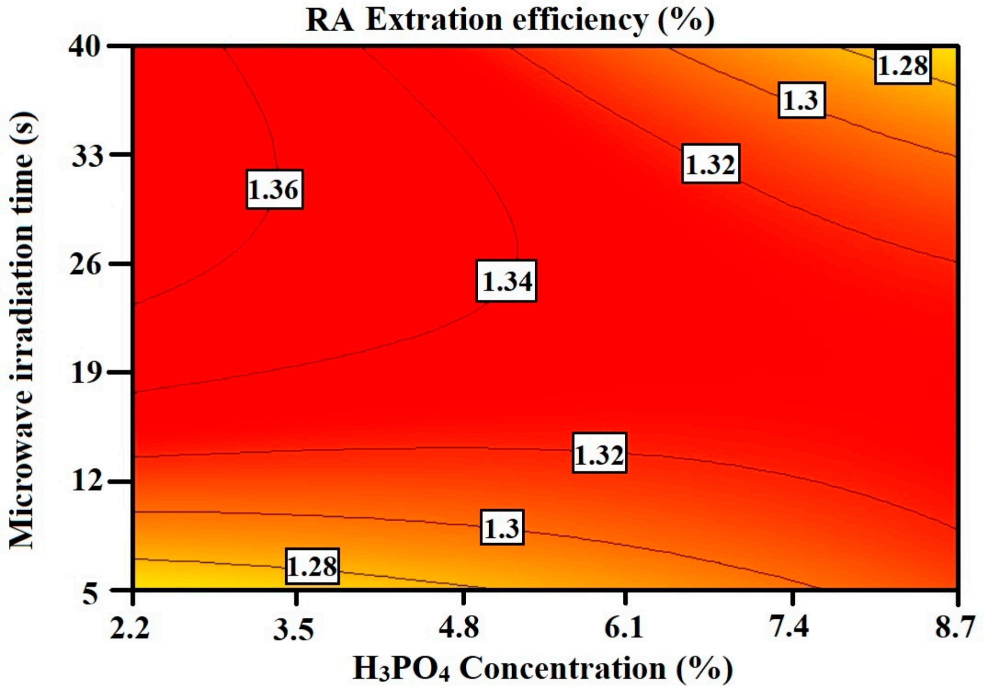

Figure S10. Contour plot of the interactive effects of H3PO4 concentration and microwave irradiation time on the extraction of RA.

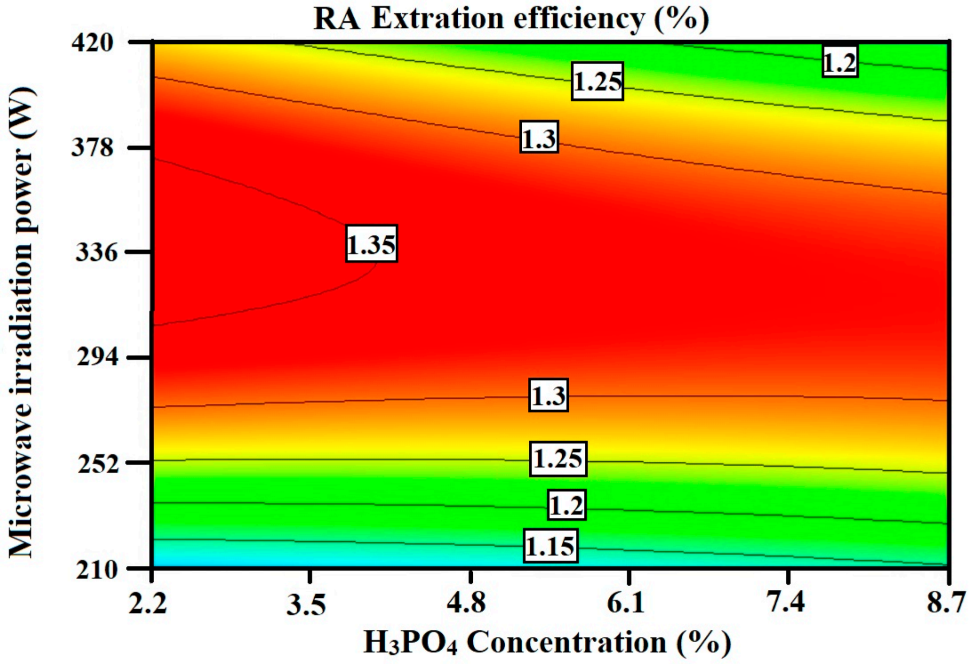

Figure S11. Contour plot of the interactive effects of H3PO4 concentration and microwave irradiation power on the extraction of RA.

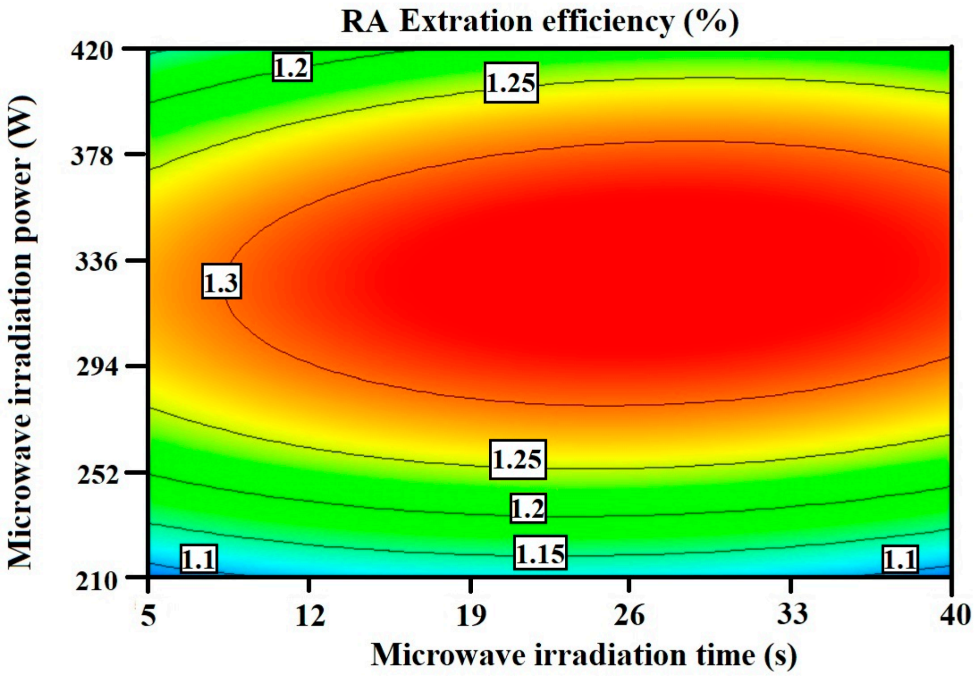

Figure S12. Contour plot of the interactive effects of microwave irradiation time and microwave irradiation power on the extraction of RA.
